# DNA Damage after Continuous Irradiation: Yanch and Engelward Respond

**DOI:** 10.1289/ehp.1205564R

**Published:** 2012-10-01

**Authors:** Jacquelyn Yanch, Bevin Engelward

**Affiliations:** 1 Department of Nuclear Science and Engineering, Massachusetts Institute of Technology, Cambridge, Massachusetts, E-mail: jcyanch@mit.edu; 2 Department of Biological Engineering, Massachusetts Institute of Technology, Cambridge, Massachusetts

We thank Beyea for his comments and would like to respond, in particular, regarding the works he cites in his letter. First, the results of our study are, in fact, consistent with the findings of many human epidemiologic studies. The latest National Research Council (NRC) report on the *Health Risks from Exposure to Low Levels of Ionizing Radiation: BEIR VII Phase 2* (NRC 2006) summarized the conclusions of studies examining cancer mortality in those occupationally exposed to long-term low dose-rate radiation (Tables 8.3–8.5). Of the 38 studies listed, approximately half (18) found either no association or a negative relationship whereby exposure to radiation correlated with a reduced cancer mortality rate.

A significant shortcoming of many of the studies listed in BEIR VII is the *a priori* invocation of the linear no-threshold (LNT) model without consideration of other plausible dose–response relationships. Because large cumulative doses do result in excess cancer deaths, fixing the lowest data point at (0,0) and assuming a linear relationship has the inevitable consequence of generating a positive dose response at all lower doses, regardless of whether this conclusion is supported by low-dose data. For instance, Table 5 of the study by Krestinina et al. (2007), cited by Beyea, shows the number of person-years represented by subjects in various dose cohorts (< 10, < 50, < 100, < 300, and ≥ 300 mGy), and the estimated number of excess cancer deaths. Note that the number of excess cancers per person-year of exposure initially declines and increases significantly only for the very highest dose cohort—those receiving any cumulative dose > 300 mGy (Krestinina et al. 2007). Our data are thus consistent with the data of Krestinina et al. (2007) but not with their conclusion, which is based on the LNT model.

Cardis et al. (2005) pooled results from nuclear workers in 15 countries; in calculating country-specific excess relative risk (ERR) per sievert, they found one country, Canada, to have an ERR > 6 times the 15-country average. As pointed out by Krestinina et al. (2007), other analyses of the Canadian cohort determined an ERR that was much lower, on the order of 2.5/Sv. If this value had been used by Cardis et al. (2005), their estimate of the ERR from the pooled cohort would not be significant because the ERR for other countries in the pool was < 0.0. Indeed, Cardis et al. stated that when they removed the Canadian cohort (which contributed only 5% of the deaths in the study), the calculated ERR for the entire group was not significantly different from zero, even though the LNT was applied. That is, exposure to radiation was found to have no impact on cancer mortality rates, a result consistent with our study, in which we found no association between radiation exposure and end points commonly associated with cancer induction.

In another study cited by Beyea, Nair et al. (2009) found an ERR no different from zero. Radiation workers typically receive occupational doses that are substantially less than their natural background doses [in the United States, occupational doses are about 30% of average natural background doses (U.S. Nuclear Regulatory Commission 2012); thus, any association between occupational radiation dose and excess cancer mortality would be very difficult to discern. In contrast, in the study by Nair et al. (2009), the subjects who lived in an area with naturally high background radiation received average radiation doses 8 times greater than the average occupational doses reported by Cardis et al. (2005). Not only did Nair et al. calculate an ERR of less than zero, the upper 95% confidence level was less than the ERR calculated from A-bomb survivor studies (Nair et al. 2009); this result is consistent with a significant dose-rate effect whereby the effects of a dose received slowly over time are substantially reduced relative to the same dose received acutely.

Muirhead et al. (2009) found that the ERR per sievert was positive for 19 types of cancer and negative for 10. These mixed results echo those of epidemiologic studies of cancers in humans exposed to ionizing radiation in general: Some show a positive relationship, some show no effect, and some show a negative correlation.

Once the environment has been contaminated with radionuclides and our dose-rate increases, how we deal with the problem is a “zero-sum proposition.” That is, to avoid additional radiation dose (beyond natural background), it is necessary to relinquish many important aspects of life as a result of evacuation and long-term relocation: homes, communities, employment, and school opportunities, among others. One question is critical: At what dose-rate should these aspects of life be relinquished for years, perhaps forever? To answer such an important question, we need to begin relying on data and not on hypothetical models that, although offering mathematical simplicity, do not reflect the complexity of a biological system that evolved on a naturally radioactive earth with exposure levels that vary considerably from place to place.
